# Acute Necrotizing Encephalitis in Viral Respiratory Tract Infection: An Autopsy Case Report

**DOI:** 10.7759/cureus.8070

**Published:** 2020-05-12

**Authors:** George S Stoyanov, Emran Lyutfi, Deyan L Dzhenkov, Lilyana Petkova

**Affiliations:** 1 General and Clinical Pathology/Forensic Medicine and Deontology, Medical University of Varna, Varna, BGR; 2 Neurology and Neuroscience, Medical University of Varna, Varna, BGR

**Keywords:** acute necrotizing encephalitis, respiratory tract infections, pathology, morphology

## Abstract

Acute necrotizing encephalitis (ANE) is a rare complication of viral respiratory tract infections, with specific histological changes. The condition is most commonly described in the pediatric population, however, it can also develop in the elderly, with some genetic factors being described as contributory. Herein, we report the autopsy finding of a patient with a viral respiratory tract infection, complicated with ANE. The patient was a 77-year-old female with multiple comorbidities living in a social home. For the two months prior, she had been hospitalized with cerebral infarction, respiratory tract infection, and exacerbation of chronic cardiac failure and concomitant hypertension and type 2 diabetes. On gross examination, the brain was edematous, with ground-glass opacity meninges a focus of encephalomalacia in the right cerebral hemisphere and multiple petechial hemorrhages. Histology revealed diffuse foci of encephalitis, with large areas of neuronal necrosis (coagulative-like necrosis) around the blood vessels and a sharp border with the surrounding healthy parenchyma - ANE. The patients tested negative for coronavirus disease 2019 (COVID-19).

## Introduction

Acute necrotizing encephalitis (ANE) is a rare complication of viral infections, most commonly described in the pediatric population [1]. As of the first quarter of 2020, multiple reports have emphasized the condition and its association with respiratory tract infections, namely, coronavirus disease (COVID-19) [2-3]. Whilst the radiological characteristics of the condition are well-defined, the histological picture is seldom discussed, which can pose a difficulty in autopsies of patients with the condition, especially if there is insufficient medical documentation on the patient’s condition [4]. Herein, we report the histological finding of a patient with a viral respiratory tract infection complicated by ANE.

## Case presentation

The patient was a 77-year-old caucasian female with multiple comorbidities living is a social home. Two months prior, she had been subsequently hospitalized with cerebral infarction, respiratory tract infection, and exacerbation of chronic cardiac failure. Concomitant diseases included hypertension and type 2 diabetes. Upon the current admission, the patient was with severe neurological deficiency progressing to a coma, exacerbated cardiac failure, and early signs of acute kidney failure. Despite the intervention performed, the patient died and was referred for an autopsy. The autopsy was video documented.

**Figure 1:**
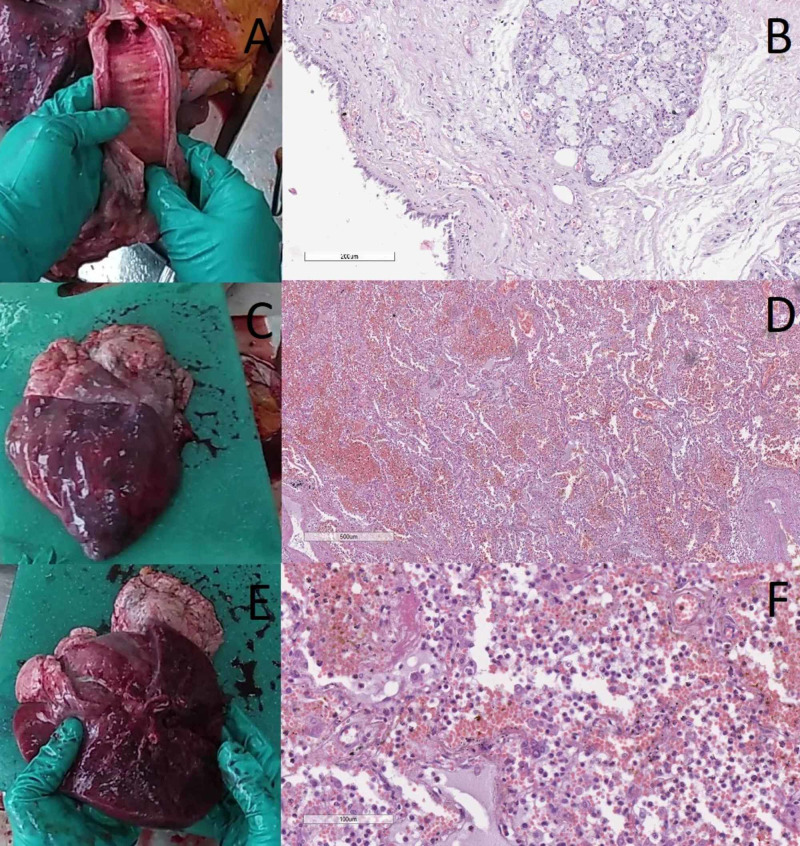
Gross and histological changes in the trachea and lung A: gross view of the trachea; B: histology of the trachea with desquamation of the respiratory epithelium, hyperemia, lymphocytic infiltrate and mucous hyperproduction, hematoxylin and eosin stain, original magnification x100; C: gross view of the lung; D: lung histology with hemorrhagic pneumonia, hematoxylin and eosin stain, original magnification x40; E: gross view of the lung on a cross-section; F: lung histology with hemorrhagic pneumonia, hematoxylin and eosin stain, original magnification x200. Note: gross images are obtained from the video recording

**Figure 2:**
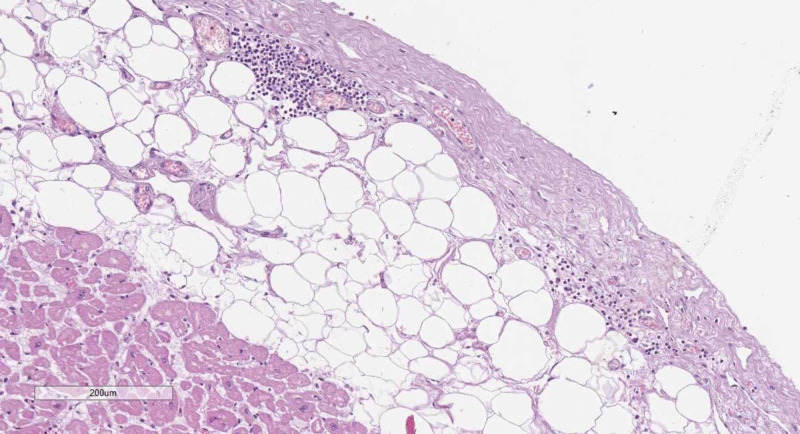
Epicardial histology Epicardium with fibrosis and focal infiltration by lymphocytes, hematoxylin and eosin stain, original magnification 100x

The section of the thoracic complex revealed edematous mucosa of the trachea and principal bronchi with multiple petechial hemorrhages and erosion, histologically correlating with serous tracheobronchitis with viral etiology (Figures 1A-1B). The left lung was grossly normal (380 g), whilst the right lung (940 g) had an upper lobe that was livid and consolidated, histologically showing hemorrhagic-purulent pneumonia (Figures 1C-1F). The vascular tree of the lung was unobstructed. The pericardium was semitransparent and slimy, with pericarditis being observed on histology (Figure 2).

**Figure 3:**
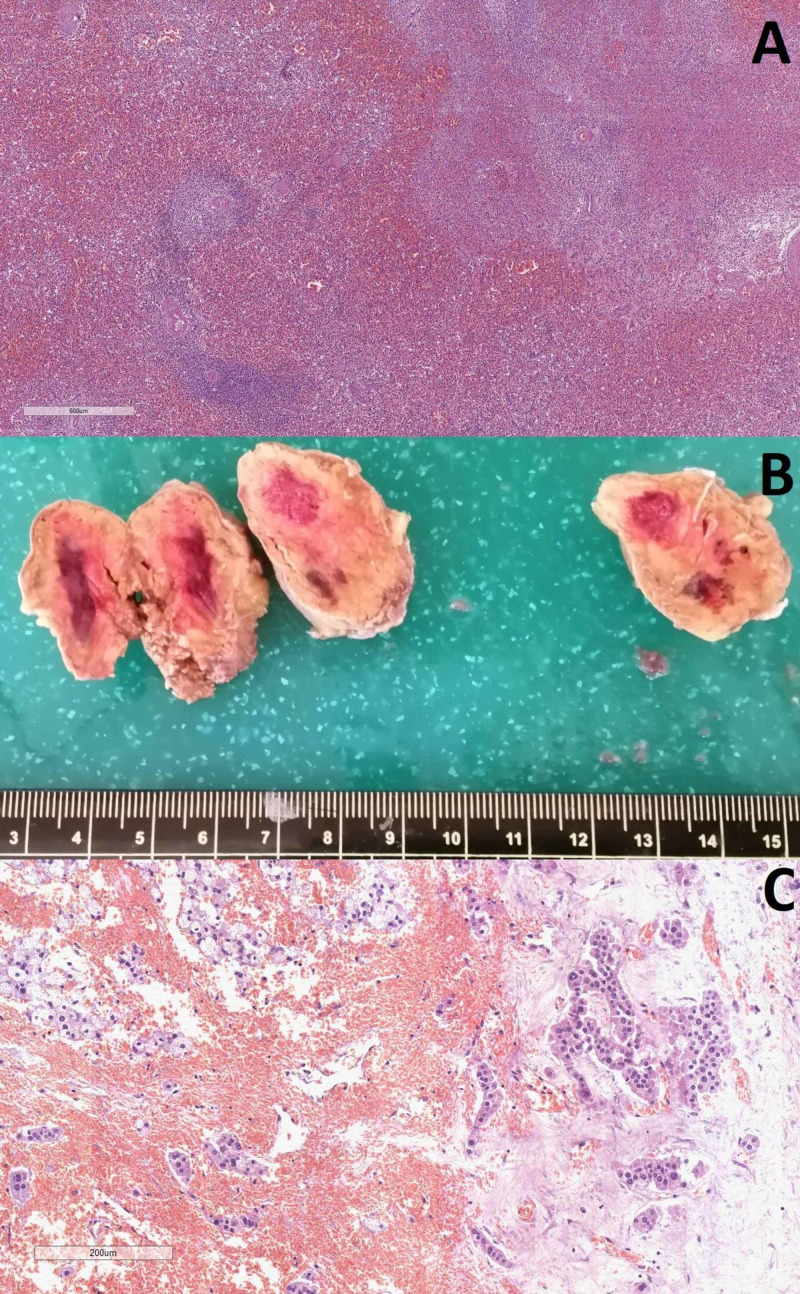
Hemorrhagic changes in the spleen and liver A: hemorrhagic areas in the spleen, hematoxylin and eosin stain, original magnification 40x; B: gross changes in the adrenal gland; C: histology from the adrenal glands with hemorrhagic areas, hematoxylin and eosin stain, original magnification 100x

The section of the abdominal organs revealed bilateral hemorrhages in the adrenal glands and petechial hemorrhages in the spleen (160 g) (Figure 3).

**Figure 4:**
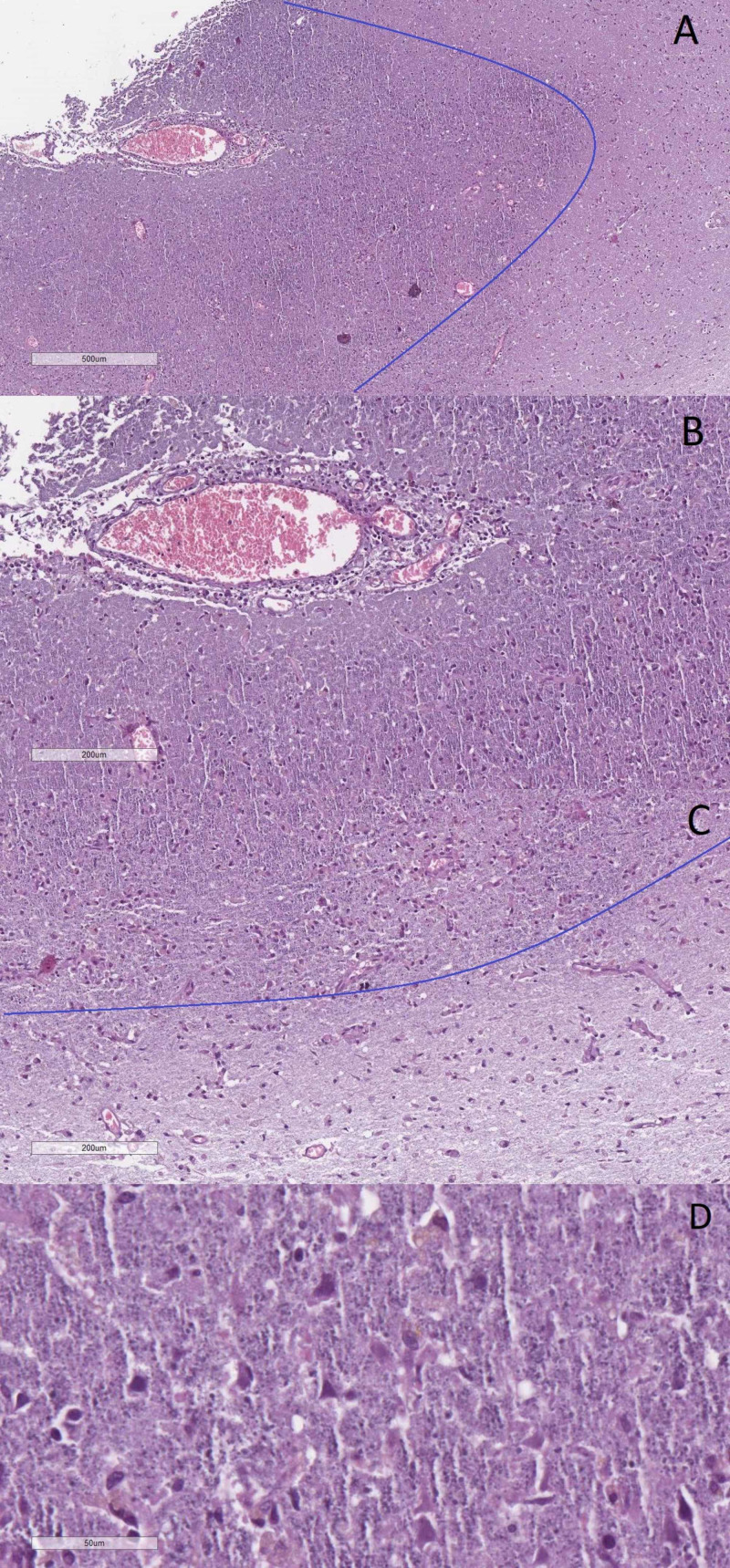
Acute necrotizing encephalitis on histology A: acute necrotizing encephalitis with neuronal necrosis (coagulative-like necrosis), neurophagia and reactive gliosis, Hematoxylin and eosin stain, original magnification 40x; B: higher magnification view of the perivascular changes with lymphocytic infiltration and degenerative and necrotic neurons, hematoxylin and eosin stain, original magnification 100x; C: the border with healthy brain parenchyma, Hematoxylin and eosin stain, original magnification 100x; D: neuronal necrosis with cellular debris and scant inclusions, hematoxylin and eosin stain, original magnification 400x. Note: blue lines on A and C depict the border between the necrotic tissue and the healthy parenchyma

The brain was edematous, with ground-glass opacity meninges a focus of encephalomalacia in the right cerebral hemisphere and multiple petechial hemorrhages. Histology revealed diffuse foci of encephalitis (inflammatory cuffs), with large areas of neuronal necrosis (coagulative-like necrosis), neurophagia, and reactive gliosis around the blood vessels and a sharp border with the surrounding healthy parenchyma - ANE (Figure 4).

**Figure 5:**
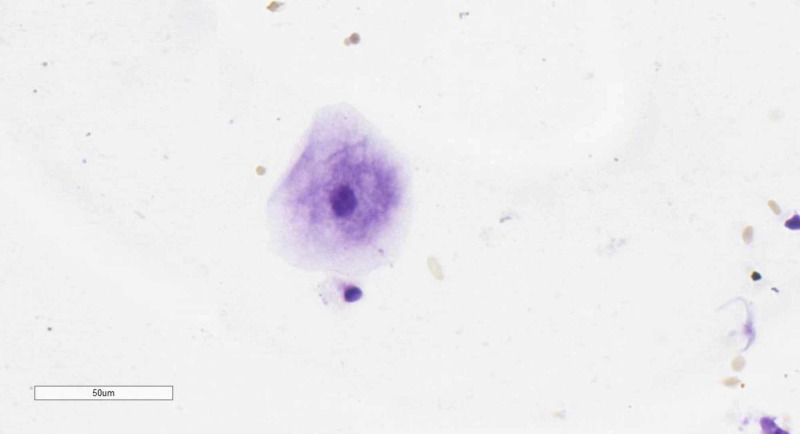
Pharyngeal smear cytology Excessive asymmetrical perinuclear granulations in superficial squamous epithelial cells from the pharynx, modified Nissl’s stain (Cresyl violet), original magnification 400x

During the autopsy based on the gross finding and the epidemiological situation with COVID-19, both a pharyngeal swab and venous blood were taken for a real-time polymerase chain reaction (PCR) test. A second pharyngeal swab was performed for cytology, stained with a one-minute protocol for Nissl’s stain (Cresyl violet) - a nucleic acid-specific stain, which revealed asymmetrical granulation in the perinuclear area of the superficial squamous epithelial cells (Figure 5). Both real-time PCR tests came back negative.

The protocol was finalized as acute respiratory infection complicated with serous tracheobronchitis, hemorrhagic pneumonia, pericarditis, leptomeningitis, acute necrotizing encephalitis, and bilateral hemorrhages in the medulla of the adrenal glands and spleen.

## Discussion

ANE is a rare complication of viral infections such as influenzas type A and B, human herpesvirus 6, and, as recently depicted, COVID-19 [1-4]. This rare complication has seldom been described in adults, such as in our case, and most cases have been described in Asian populations [1,5].

There are also several genetic factors with an established contributory role to the development of ANE [5]. The clinical outcome can often be fatal, as in our case, however, there have been cases with full neurological recovery.

Morphologically the type of necrosis seen is much more similar to coagulative necrosis, which has only been associated with several causative agents such as toxoplasma in the central nervous system (CNS), drastically different to the liquefactive necrosis observed in CNS ischemia. This morphology suggests direct neuron-specific action of the pathogen, such as pyrexia or neuronal toxicity, rather than non-specific pan-cellular damage such as ischemia. Coupled together with the specific genetic pattern, this could present as genotype-specific neuronal damage in viral infections only in susceptible individuals [5].

So far, there have been only a few case reports or series of ANE with histology in humans [6-8]. The neuronal necrosis seems to affect predominantly the cortex in a patchy manner, with the temporal lobe being the one affected most often [6-8]. This would easily explain the cognitive deficit recently described in COVID-19 patients with ANE on presentation [2-3]. A secondary location commonly affected are also the basal ganglia, again in a non-diffuse patchy manner [6-8].

## Conclusions

ANE is a rare complication of viral infections, presenting morphologically with encephalitis and scattered cortical, predominantly in the temporal lobe and basal ganglia, foci of neuronal necrosis, neurophagia, and reactive gliosis in the brain parenchyma. There have been several viruses described that can lead to ANE, with genetic as well as age-related predisposing factors also playing a role.

## References

[1] Goenka A, Michael BD, Ledger E (2014). Neurological manifestations of influenza infection in children and adults: results of a National British Surveillance Study. Clin Infect Dis.

[2] Jebril N (2020). Viral encephalitis associated with COVID-19: a review of the literature and two cases. SSRN Electron J.

[3] Poyiadji N, Shahin G, Noujaim D, Stone M, Patel S, Griffith B (2020). COVID-19-associated acute hemorrhagic necrotizing encephalopathy: CT and MRI features. Radiology.

[4] Ochi N, Takahashi K, Yamane H, Takigawa N (2018). Acute necrotizing encephalopathy in an adult with influenza A infection. Ther Clin Risk Manag.

[5] Suri M (2010). Genetic basis for acute necrotizing encephalopathy of childhood. Dev Med Child Neurol.

[6] Bennett DR, Zurhein GM, Roberts TS (1962). Acute necrotizing encephalitis. A diagnostic problem in temporal lobe disease: report of three cases. Arch Neurol.

[7] Adams JH, Jennett WB (1967). Acute necrotizing encephalitis: a problem in diagnosis. J Neurol Neurosurg Psychiat.

[8] Ishii N, Mochizuki H, Moriguchi-Goto S (2015). An autopsy case of elderly-onset acute necrotizing encephalopathy secondary to influenza. J Neurol Sci.

